# Type I Collagen Synthesis Marker Procollagen I *N*-Terminal Peptide (PINP) in Prostate Cancer Patients Undergoing Intermittent Androgen Suppression

**DOI:** 10.3390/cancers3033601

**Published:** 2011-09-15

**Authors:** Gerhard Hamilton, Ulrike Olszewski-Hamilton, Gerhard Theyer

**Affiliations:** 1 Ludwig Boltzmann Cluster of Translational of Oncology, Nussdorfer Strasse 64, Vienna A-1090, Austria; E-Mail: ulrike.olszewski@toc.lbg.ac.at; 2 Hospital Kittsee, Kittsee A-2421, Burgenland, Austria; E-Mail: gerhard@theyer.at

**Keywords:** intermittent androgen suppression, prostate cancer, prostate-specific antigen, bone turnover, PINP

## Abstract

Intermittent androgen suppression (IAS) therapy for prostate cancer patients attempts to maintain the hormone dependence of the tumor cells by cycles alternating between androgen suppression (AS) and treatment cessation till a certain prostate-specific antigen (PSA) threshold is reached. Side effects are expected to be reduced, compared to standard continuous androgen suppression (CAS) therapy. The present study examined the effect of IAS on bone metabolism by determinations of serum procollagen I *N*-terminal peptide (PINP), a biochemical marker of collagen synthesis. A total of 105 treatment cycles of 58 patients with prostate cancer stages ≥pT2 was studied assessing testosterone, PSA and PINP levels at monthly intervals. During phases of AS lasting for up to nine months PSA levels were reversibly reduced, indicating apoptotic regression of the prostatic tumors. Within the first cycle PINP increased at the end of the AS period and peaked in the treatment cessation phase. During the following two cycles a similar pattern was observed for PINP, except a break in collagen synthesis as indicated by low PINP levels in the first months off treatment. Therefore, measurements of the serum PINP concentration indicated increased bone matrix synthesis in response to >6 months of AS, which uninterruptedly continued into the first treatment cessation phase, with a break into each of the following two pauses. In summary, synthesis of bone matrix collagen increases while degradation decreases during off-treatment phases in patients undergoing IAS. Although a direct relationship between bone matrix turnover and risk of fractures is difficult to establish, IAS for treatment of biochemical progression of prostate tumors is expected to reduce osteoporosis in elderly men often at high risk for bone fractures representing a highly suitable patient population for this kind of therapy.

## 1. Introduction

Prostate cancer is among the most common types of malignancies and causes of cancer-related deaths in men worldwide. Patients with tumors, which are advanced at presentation or relapsed following radical prostatectomy have a dismal prognosis [1,2]. Treatment traditionally consists of androgen suppression (AS) of the growth of cancer cells either by orchidectomy or the use of LHRH analogs and steroidal or nonsteroidal antiandrogens, respectively [3]. AS is conventionally performed in a continuous regimen and results in apoptotic regression of the tumors in most cases. However, continuous androgen suppression (CAS) controls tumor growth for only two to three years until hormone-resistant cancers, which respond poorly to any further therapy including treatment with chemotherapeutics, appear [4,5]. Therefore, intermittent androgen suppression (IAS) was proposed as a novel clinical concept assuming that tumorigenic stem cells are residing in an androgen-sensitive state during limited regrowth in treatment cessation periods [6,7]. Since then, regrowing tumors in patients undergoing IAS were consistently shown to be sensitive over several cycles of androgen withdrawal and this kind of therapy resulted in improved quality of life [7-9]. Meanwhile, phase III studies established IAS as therapy equivalent to CAS in respect to survival leading to the proposal of IAS as standard therapy for progressive prostate cancer [10,11].

Besides other side effects, CAS results in increased incidence of osteoporosis and concomitant bone fractures [12,13]. Therefore, it was expected that off-treatment periods of IAS would allow for recovery of testosterone levels and cessation of bone matrix degradation. Higano *et al.* observed that loss of bone matrix density (BMD) after nine months of AS was significantly greater than the expected 0.5–1% annual decrease; however, interruption of AS attenuated the rate of bone degradation without full recovery and other clinical studies were inconclusive [14,15]. Apart from clinical assessments of BMD no reports of biochemical studies on bone metabolism were available prior to our publication on collagen degradation products. In analogy to other bone matrix turnover investigations of we quantified the degradation using the serum marker CrossLaps^®^ in IAS patients and found increased breakdown at the end of the AS phase in contrast to reduced anabolism during the treatment cessation period [16]. In order to assess synthesis of bone matrix, levels of PINP, a maturation peptide of collagen I, were retrospectively analyzed in serum samples retrospectively in the present study to obtain a more complete characterization of bone turnover during IAS [17].

## 2. Results

### 2.1. Characteristics of IAS Cycles in Prostate Cancer Patients

All patients (n = 58; median age: 69 years, range: 53–82 years) exhibited disease progression following radical prostatectomy and/or irradiation therapy. Lengths of treatment cessation periods (mean ± SEM) for the respective off-treatment phases (PI–PIV) were in months: 14.8 ± 1.4 (n = 58), 10.9 ± 1.8 (n = 29), 7.5 ± 1.0* (n = 14) and 8.2 ± 1.4 (n = 4), respectively. The first treatment cessation period (PI) tended to be longer than the subsequent pauses; however, the single statistically significant difference in duration was between PI and PIII.

### 2.2. Individual Course of Testosterone and PINP Levels under IAS

**Figure 1.**
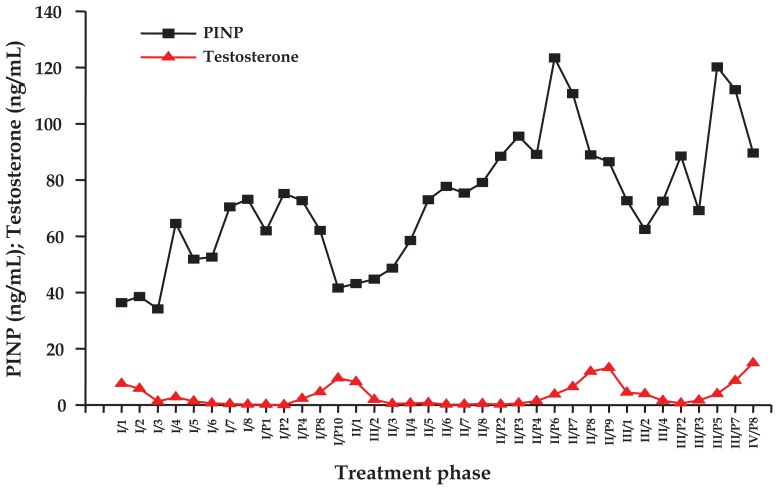
Time courses of testosterone and PINP levels during three IAS cycles in a prostate cancer patient. I–III indicate the three AS and I/P–III/P the treatment cessation periods, with arabic characters denoting the respective month of observation.

Individual time courses of concentrations of testosterone and PINP for a representative patient undergoing IAS are depicted in Figure 1. The figure shows the values of the laboratory parameters for cessation and treatment periods, respectively, which were measured in monthly intervals. Testosterone repeatedly dropped during the AS periods and recovered significantly during the treatment breaks. Concentrations of PINP began to rise between three to four months of AS and showed a decrease, which was incomplete during treatment cessation periods and revealed lowest values concomitant with the respective testosterone peaks.

### 2.3. Mean Course of PSA under IAS

**Figure 2.**
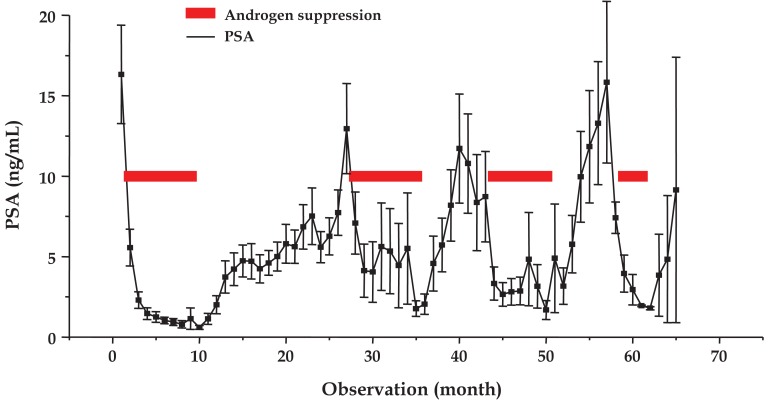
Mean time course of PSA during four IAS cycles of the prostate cancer patient group in total (observations monthly, mean values ± SEM). Red bars indicate the four AS periods.

The mean time course of serum concentrations of PSA (mean ± SEM) for a total of 105 IAS cycles observed in 58 patients is shown in Figure 2. AS triggered decreases of testosterone to values <1 ng/mL, which was followed by recovery to baseline levels in the third month of treatment cessation (data not shown). During AS all patients showed reversible declines in PSA production to a mean level of <2 ng/mL for four cycles. Treatment cessation led to reappearance of PSA for four cycles to 16.3 ± 3.1, 13.0 ± 2.8, 10.8 ± 3.1 and 15.8 ± 5.0 ng/mL, respectively. Since some observations are lacking for the treatment cessation periods, the duration of the cycle lengths specified in Section 2.1 is actually longer than presented in Figure 2.

### 2.4. Mean Course of PINP under IAS

**Figure 3.**
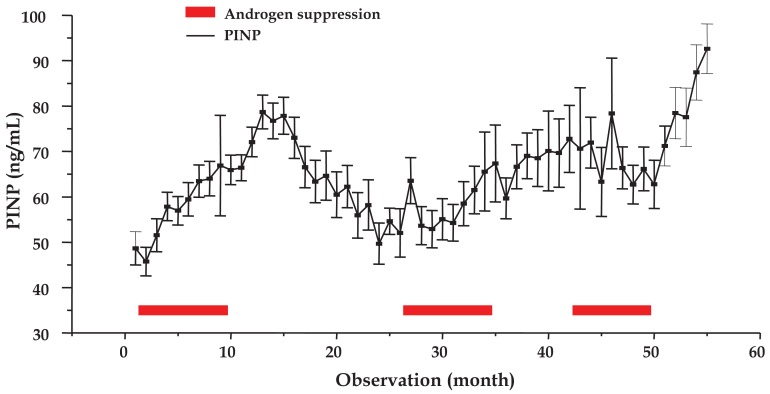
Mean time course of PINP levels over three IAS cycles of the prostate cancer patient group in total (observations monthly, mean values ± SEM). The three AS periods are indicated by red bars.

The mean time course of PINP concentrations analyzed for a total of 105 cycles of 58 patients is shown in Figure 3 (mean ± SEM). Measurements for observations 7–17, 28, 35–39 (except 36) and 52–55 were significantly different from the pretreatment value (P < 0.05). Therefore, bone matrix anabolism increased three months before the first AS period ended and peaked in the treatment cessation period (months 4–6) prior to a return to a minimum just ahead of the second AS phase. Further significant elevations were observed during the second AS phase (months 2 and 9) as well as in the following treatment pause (months 4–9) and, finally, during the third treatment cessation period (months 2–5).

## 3. Discussion

Advanced stage adenocarcinoma of the prostate is treated by surgical and/or antiandrogenic hormone ablation [2,18]. CAS provides selective pressure on the tumor cells, invariably resulting in outgrowth of variants adapted to very low androgen concentrations or relying on androgen-independent proliferative stimuli [19]. In contrast, IAS attempts to prolong the hormone dependence of tumor cells by allowing for limited regrowth of hormone-sensitive cells between suppression periods to hold the tumor at bay [6,7]. Phase III studies provide solid evidence that IAS is not inferior to CAS in terms of survival for selected patient groups, albeit hormone dependence of the tumor cells may not be lengthened [10,20]. Assuming equipotency of these therapies, the nature and severity of side effects and costs of each regimen will be decisive for its clinical use.

In detail, adverse effects of CAS include skeletal, metabolic and cardiovascular complications, sexual dysfunction, hot flashes as well as cognition and mood disorders [13]. In particular, it was demonstrated that CAS reduced BMD, which led to increased risk of skeletal fractures [12,21]. In the largest study of men receiving CAS (390 patients), the prevalence of osteoporosis was 35% in hormone-naïve patients, 43% after two years of CAS and 81% after ten or more years [22]. Several groups investigated the effects of IAS on BMD and reported reduction of bone loss upon prolonged treatment. Higano *et al.* described increased bone loss during the AS phase of IAS and partial recovery during cessation [14]. Spry *et al.* reported significant improvement of hip BMD following two years of IAS, which was dependent on testosterone recovery [15]. Malone *et al.* did not notice any increase of osteoporosis in patients under IAS compared to data from age-matched individuals without prostate cancer [23]. Hence, from the clinical measurements of BMD in limited groups of IAS patients the reversal of AS-induced bone loss during the off-treatment periods is still not clear.

The effect of IAS on BMD may be studied quantitatively using biochemical markers of bone metabolism, as far as there is no interference from bone metastatic lesions [17,24]. Collagen I accounts for more than 90% of the organic matrix of bone and is synthesized by osteoblasts and degraded by osteoclasts during remodeling. Crosslinked degradation telopeptide fragments of collagen I can be measured by a CrossLaps^®^ ELISA [25,26]. We recently published collagen degradation, marked by elevated levels of CrossLaps^®^ at the end of the AS phases, was reduced during the treatment cessation periods of IAS below pretreatment concentrations [16]. Synthesis of collagen can be assessed through measurements of PINP, which is cleaved from newly formed procollagen chains [27,28]. Surprisingly, the first AS cycle stimulated collagen production during the last months of hormone ablation and this effect continued well into the first treatment cessation period until it returned to baseline levels prior to the next AS phase. Sporadic elevations of PINP were detected during the subsequent AS periods followed by significant increases during off-treatment phases in the second and third IAS cycle. This combination of decreased degradation of collagen I, as indicated by measurements of CrossLaps^®^ and increased production, proved by quantitation of PINP, is expected to limit bone matrix anabolism and reduce AS-induced osteoporosis. These findings confirm the positive effects reported from other clinical IAS studies on BMD [29]. IAS seems to be most suitable for elderly men that show biochemical progression following prostatectomy and/or irradiation, likewise comprising the population at greatest risk for bone fractures. Furthermore, a significant fraction of the same group of patients exhibited prolonged responses to the first AS phase of IAS and accomplished a subsequent treatment cessation period of up to several years [30]. Limiting the exposure to AS constitutes the most simple method to reduce side effects and to avoid problems and costs associated with the medical treatment of osteoporosis by calcium/vitamin D supplementation or drugs like bisphosphonates or a monoclonal antibody [21]. Additionally, intermittent hormone deprivation may result in a reduction of further side effects, especially metabolic and cardiovascular complications [11].

## 4. Experimental Section

### 4.1. Study Population and Treatment

All patients gave written informed consent according to approval guidelines of the ethics committee. Between June 1993 and August 2003 all patients with disseminated adenocarcinoma of the prostate fulfilling the inclusion criteria of histologically confirmed tumors of stage ≥T2 not having received pretreatment with either hormone ablation or chemotherapy and rising PSA levels were recruited for our nonrandomized open IAS trial. Treatment consisted of an initial nine-months course of AS (LHRH agonist goserelin acetate/Zoladex^®^ and antiandrogen cyproterone acetate/Androcur^®^) followed by treatment cessation and resumption of the therapy as soon as PSA increased above 4 or 20 ng/mL for local or metastatic disease, respectively [31]. LHRH agonist Leuprorelin/Trenantone^®^ was used for nine months of AS from 2003 on. Follow-up examinations included digital rectal examination, transrectal sonography, yearly chest X-rays and bone scans, respectively.

### 4.2. Laboratory Measurements

Blood samples were taken from each patient prior to treatment and at monthly intervals thereafter and serum was stored at −80 °C. Serum testosterone concentrations were measured using an ELISA assay (Biomar Diagnostics, Marburg, Germany) according to the manufacturer's instructions. PSA levels were determined by a microparticulate enzyme immunoassay (MEIA, AxSYM PSA assay, Abbott, Wiesbaden, Germany) and CrossLaps^®^ ELISA was obtained from Nordic Bioscience Diagnostics (Herlev, Denmark) and used according to the manufacturer's instructions. All determinations were done in duplicate.

### 4.3. Statistical Analysis

Student's *t*-test was used for the statistical analyses. * P < 0.05 was considered statistically significant. All calculations were done using the Statistica software package (Statsoft, Tulsa, OK, USA).

## 5. Conclusions

In conclusion, measurements of serum CrossLaps^®^ levels revealed significant BMD in prostate cancer patients at the end of the AS phases of IAS cycles, which was rapidly reversed during the treatment cessation periods [16]. According to the results from our present study, tracking of the course of PINP levels shows increased synthesis of bone matrix collagen I in response to limited periods of hormone deprivation in IAS. These findings indirectly corroborate the decreased loss of BMD in bone scans in prostate cancer patients under IAS therapy, and determinations of markers of bone turnover are recommended to be performed along with bone scans in IAS patients to establish a quantitative relationship. Thus, elderly patients with a prolonged off-treatment interval and a good long-term prognosis are expected to have reduced bone losses, without additional medication under IAS therapy [21,30].
